# Validity and reliability of the Mexican resilience measurement scale in families of children with chronic conditions

**DOI:** 10.1186/s12955-017-0817-3

**Published:** 2017-12-13

**Authors:** Filiberto Toledano-Toledano, José Moral de la Rubia, Laurie D. McCubbin, Linda Liebenberg, Jesús Alejandro Vera Jiménez, Leonor Rivera-Rivera, Angie Hart, Leticia Andrea Barajas Nava, Marcela Salazar García, Silvia Martínez Valverde, Sofía Rivera Aragón, Concepción Sánchez Gómez, Laura Villavicencio Guzmán, Victor Granados García, Juan Garduño Espinosa

**Affiliations:** 1 Unidad de Investigación en Medicina Basada en Evidencias, Hospital Infantil de México Federico Gómez Instituto Nacional de Salud, Dr. Márquez 162, Doctores, Cuauhtémoc, 06720 México City, Mexico; 2 Facultad de Psicología, Universidad Autónoma de Nuevo León, Dr. Carlos Canseco, 110, Esq. Dr. Aguirre Pequeño, Col. Mitras Centro, 64460 Monterrey, Mexico; 3 College of Education and Human Development, University of Louisville, 1905 S 1st St, Louisville, KY 40208 USA; 4 Faculty of Graduate Studies, Dalhousie University, PO Box 15000, Halifax, NS Canada; 5 Centro de Investigación Transdiciplinar en Psicología, Universidad Autónoma del Estado de Morelos, Pico de Orizaba 1. Col. los Volcanes, 62350 Cuernavaca, Morelos Mexico; 6 Centro de Investigación en Salud Poblacional, Instituto Nacional de Salud Pública, Av. Universidad No. 655 Col. Santa María Ahuacatitlán, 62100 Cuernavaca, Morelos Mexico; 7 School of Health Sciences, University of Brighton, 264 Mayfield House, Falmer, East Sussex, BN1 9PH UK; 8 Laboratorio de Investigación en Biología del Desarrollo, Hospital Infantil de México Federico Gómez, Instituto Nacional de Salud, Dr. Márquez 162, Doctores, Cuauhtémoc, 06720 México City, Mexico; 9 Centro de Estudios Económicos y Sociales en Salud, Hospital Infantil de México Federico Gómez Instituto Nacional de Salud, Dr. Márquez 162, Doctores, Cuauhtémoc, 06720 México City, Mexico; 10 Facultad de Psicología, Universidad Nacional Autónoma de México, Avenida Universidad 3004, Copilco Universidad, Coyoacán, 04510 México City, Mexico; 11 Unidad de Investigación Epidemiológica y en Servicios de Salud, Área Envejecimiento. 3er piso. Edificio CORSE, Centro Médico Nacional Siglo XXI. Av. Cuauhtémoc 330. Doctores Cuauhtémoc, 06720 México City, Mexico; 12 Dirección de Investigación, Hospital Infantil de México Federico Gómez Instituto Nacional de Salud, Dr. Márquez 162, Doctores, Cuauhtémoc, 06720 México City, Mexico

**Keywords:** Validity, Reliability, Resilience, Pediatric chronic disease, Family caregivers, Psychometric properties, Instrumental study, Mexican version, RESI-m

## Abstract

**Background:**

The resilience to face disease is a process of positive adaptation despite the loss of health. It involves developing vitality and skills to overcome the negative effects of adversity, risks, and vulnerability caused by disease. In Mexico, the Mexican Resilience Measurement Scale (RESI-M) has been validated with a general population and has a five-factor structure. However, this scale does not allow evaluation of resilience in specific subpopulations, such as caregivers.

**Method:**

This study investigated the psychometric properties of RESI-M in 446 family caregivers of children with chronic diseases. A confirmatory factor analysis (CFA) was performed, internal consistency values were calculated using Cronbach’s alpha coefficient, and mean comparisons were determined using t-tests.

**Results:**

The expected five-factor model showed an adequate fit with the data based on a maximum likelihood test. The internal consistency for each factor ranged from .76 to .93, and the global internal consistency was .95. No average difference in RESI-M and its factors was found between women and men.

**Conclusion:**

The RESI-M showed internal consistency and its model of five correlated factors was valid among family caregivers of children with chronic diseases.

## Background

The resilience to face disease is a process of positive adaptation despite the loss of health. It involves developing vitality and skills to overcome the negative effects of adversity, risks, and vulnerability caused by the disease. Resilience has been defined in multiple ways [1–5], although one aspect common to all of them is the ability to adapt and achieve optimal functioning in the face of unfavorable conditions that pose risks and threaten one’s integrity. Several studies have shown that overcoming a significant loss or a potentially traumatic event results in increased resilience and therefore greater adaptability to adverse situations [6, 7]. Given the implications for the prevention of mental health problems [8, 9] and promoting human development [10], this construct has been an object of theoretical and empirical research in recent decades [11–13], particularly in positive psychology [14] and psychometry [15].

In the field of psychometry, several instruments have been developed to measure resilience [16–18]. One of these is the 25-item Connor-Davidson Resilience Scale (CD-RISC) [19], which is among the instruments with the most robust psychometric properties [12, 20]. This scale in its original version or in its short version with 10 items (CD-RISC10) [21] has been validated for use in populations of eastern [9, 14, 22] and western non-English speaking societies [23–25]. For the Mexican population, the CD-RISC [19], in addition to the Resilience Scale for Adults (RSA) [17], was the basis for the development of the Mexican Scale of Resilience Measurement (RESI-M) [26]. The RESI-M was validated for use in a general population of students and employees, of both sexes and with an age range of 18 to 25 years. It consists of a Likert scale instrument composed of five factors: strength and self-confidence, social competence, family support, social support, and structure, each with high internal consistency values (from .79 to .92). The overall internal consistency was also high (alpha = .93), and the five factors extracted accounted for 43.60% of the total variance. Sociodemographic differences were reported in the social support factor, with women and single participants scoring higher, and for strength and self-confidence, with married men scoring highest. Other research has confirmed the psychometric properties of RESI-M and the stability of its factors in a subpopulation determined by the geographic area of the country [27]. In subsequent studies, RESI-M was used to evaluate resilience in primary caregivers with normal or complicated grief, the latter characterizing those showing less possession of this ability [28], and to demonstrate resilience as an important variable in the grieving process of women with cancer [29].

Although there is evidence with respect to the adequate psychometric properties of RESI-M [26, 27], it must be considered that this scale was originally validated for use in the general population, so its use in other populations compromises the validity of its results. There is evidence that a validated scale with a general population presents a different factor structure when validated with a population of caregivers [30], results that undermine those obtained by Ornelas-Tavares [28]. In the same way, a scale validated with a general population may produce inaccurate data when applied to a clinical population [15], as the study of Miaja and Moral [29] shows. This situation can be resolved by making validations ex professo for the population to be investigated. For example, the CD-RISC10, validated in Spanish with university students [31], was later validated with patients with fibromyalgia [32] to guarantee the validity of the results in each population.

In Mexico, to date, no studies have validated the RESI-M or some other scale used to measure resilience [33, 34] in the caregiver population. This is despite the fact that the activities they carry out make them a vulnerable population [35], prone to presenting symptoms of stress, anxiety and depression [36–38] as well as experiencing a decrease in their quality of life [39, 40].

Pediatric chronic diseases represent one of the greatest challenges for family environments, with physical, psychological, socioeconomic, and behavioral effects on patients and their caregivers [41]. In addition, in recent years, the prevalence in Mexico of chronic diseases, particularly cancer [42], in children under 18 years of age has reportedly increased, which in turn has led to an increase in the number of caregivers for this population. These caregivers, usually the parents of the child patient [35], are prone to high levels of stress [43].

Given the importance of resilience for coping, focusing on problems rather than emotions [44] and on the adaptation of family members to the exigencies of chronic disease enables such individuals to face it and transcend it [45, 46]. The present study aimed to validate the five-factor model for the RESI-M in a population of family caregivers of hospitalized children with chronic diseases; calculate the internal consistency of the scale and its five factors; and compare mean scores between both sexes. The hypothesis was that the model of five correlated factors proposed by Palomar and Gomez [26] showed a good or adequate fit with the data in this population of caregivers. The following results were expected: high internal consistency value for the scale; high values for its factors, except an acceptable value for the structure factor [26]; and statistically equivalent averages in the RESI-M total score and its five factors between both sexes, except in the social support factor, in which women could have a higher average than men [26].

## Methods

### Ethical considerations

The protocol of the present study was approved by the Ethics and Biosafety Committee of the Hospital Infantil de Mexico Federico Gómez Instituto Nacional de Salud and, in its conduction, the ethical rules and considerations for research with humans currently in force in Mexico [47] as well as those outlined by the American Psychological Association [48]. The collaboration of the participants in this study was voluntary, and prior to completion, they were all informed of their rights, according to the Helsinki Declaration [49].

### Sample

The present study was conducted using a cross-section and ex post facto design [50], with non-probability sampling for convenience. A total of 446 family caregivers of children with chronic diseases hospitalized in the Children’s Hospital of Mexico, Federico Gómez, in Mexico City were recruited as subjects. The sample included women (82.3%) and men (17.7%) ranging in age from 18 to 63 years, with a mean age of 32.2 (SD = 8.7). The majority of the participants were married (40.1%) or in a free union (37.4%), with housewife as their main occupation (65.5%). The highest percentage of participants (60.5%) reported income of up to US $132 per month and primary and secondary education (63%). The inclusion criteria were 1) being 18 years of age or older, 2) being the father or mother or family caregiver of children hospitalized due to the chronic disease for which data were collected, and 3) having read and signed an informed consent form. The children cared for were girls (48%) and boys (52%) with an age range of 1 to 17 years and a mean age of 5.94 (SD = 5.07). The time elapsed since diagnosis was up to one year for most infants (67.3%) and greater than one year and up to 10 years for the others (32.7%). The most frequent diagnosis was some type of cancer (61.3%), and the time elapsed since hospitalization was from one week to one month in the majority of cases (84.3%).

#### Sample size

One rule of thumb advises including at least five participants per parameter to be estimated [51]. The number of different parameters estimated in the five-factor model was 96; therefore, based on this rule, the minimum number of participants should be 480. To the extent that the number of indicators per factor is high (6 or more), with high measurement weights (greater than .50) and few factors (five or less), a sample size larger than 400 can be judged as good [51, 52]. Therefore, the criterion to determine sample size resulted in a minimum of 400 participants and a maximum of 500 participants, that is, a sample size in an interval from 400 to 500 participants.

### Procedure

Family caregivers were contacted by the research team in the hospitalization rooms of the Hospital Infantil de México Federico Gómez, where their children received treatment. Team members then asked caregivers to participate in the study, explaining the objectives of the study and clarifying any doubts the caregivers may have. Caregivers who agreed to participate were required to sign an informed consent form and subsequently answer the RESI-M and a sociodemographic data questionnaire. The instruments were applied individually, with subjects answering questions on their own in a single session.

### Self-assessment instruments

*Socio-demographic data questionnaire*. Twenty items were used to assess sociodemographic variables related to the individual, the family, and the context of the caregiver. These variables were age, gender, marital status, years of marriage, level of studies, religion, number of children, occupation, place of origin, parental role, type of family, life cycle of the family, social support networks, and monthly family income. In addition, the questionnaire was used to obtain information related to the child: gender, age, diagnosis, medical service, time of hospitalization, and time since diagnosis.

*The Measurement Scale of Resilience in Mexicans* (RESI-M; [26]). This self-evaluation report comprises 43 four-point Likert-type items, each ranging from 1 (strongly disagree) to 4 (completely agree), distributed across five factors: 1) Strength and Self-Confidence, items 1–19, α = 0.93 (e.g., "What happened to me in the past makes me feel confident to face new challenges"); 2) Social Competence, items 20–27, α = 0.87 (e.g., "I feel comfortable around other people"); 3) Family Support, items 28–33, α = 0.87 (e.g., "I have a good relationship with my family"); 4) Social Support, items 34–38, α = 0.84 (e.g., "I have some friends/relatives who are genuinely concerned about me"); and 5) Structure, items 39–43, α = 0.79 (e.g., "Rules and routine make my life easier"). The entire instrument explains 43.6% of the total variance and has a reliability at α = .93 [26].

### Data analysis

The model of five correlated factors was tested with a confirmatory factor analysis (CFA) using the robust maximum likelihood estimation due to a deviation from normality observed in the data. Accordingly, robust standard errors and robust adjustment indexes were calculated. The goodness of fit was assessed using the Satorra-Bentler scaled chi-square (_SB_χ^2^), relative chi-square (_SB_χ^2^/*df*), Bentler’s comparative fit index (*CFI*), and root mean square error of approximation (*RMSEA*). It was stipulated that *p* > .05 for _SB_χ^2^, _SB_χ^2^/*df* ≤ 2, *IFC* ≥ .95, and *RMSEA* ≤ .05 reflected a good fit and that *p* > .01 for _SB_χ^2^, _SB_χ^2^/*df* ≤ 3, *IFC* ≥ .90, and *RMSEA* ≤ .08 reflected an acceptable fit [51]. Internal consistency values for RESI-M and its five factors were estimated using Cronbach’s alpha coefficient (α). Cronbach’s alpha coefficient is considered to indicate high internal consistency for values ≥ .80, adequate consistency for values ≥ .70, and low consistency for values < .60 [53]. Finally, Student’s t-test was performed to compare the means of the RESI-M total score and its factors between both sexes. Statistical calculations were performed using LISREL (version 6.1), the SPSS statistical package (version 22), and Excel 2007. Missing values were replaced by the arithmetic mean. The percentage of non-response was low, i.e., lower than 3% per item, with a maximum of two responses per participant.

## Results

Table 1 describes the sociodemographic characteristics of the family caregivers and children with chronic diseases in the initial sample.

**Table 1:** Sociodemographic characteristics of caregivers and children

Caregiver (*N* = 446)			Patient (*N* = 446)		
Variables	*M (SD)*	*n (%)*	Variables	*M (SD)*	*n (%)*
Age	32.23 (8.65)		Sex Women Men		214 (48) 232 (52)
Marital Status Married Living together/Co-habitation Separated Single mother Divorced Widow or widower Other		179 (40.1) 167 (37.4) 40 (9) 34 (7.6) 13 (2.9) 6 (1.3) 7 (1.6)	Age (months)	32.21 (128.81)	
Schooling No schooling Primary and secondary education High school University		15 (3.4) 281 (63) 115 (25.8) 35 (7.8)	Length of hospitalization (months)	1.71 (1.22)	
Occupation Homemaker Employee Trader Unemployed Worker Student		292 (65.5) 60 (13.5) 43 (9.6) 31 (7) 15 (3.4) 5 (1.1)	Time since diagnosis (months)	3.5 (2.00)	
Parental role Mother Father Grandmother Uncle Sibling		344 (77.1) 75 (16.1) 13 (2.9) 13 (2.9) 4 (.9)			
Family type Nuclear Single parent Semi-nuclear Extended Other		225 (50.4) 74 (16.6) 68 (15.2) 46 (10.3) 33 (7.4)			
Family life cycle With little children With school-age children With adult children		146 (32.73) 264 (59.2) 35 (7.84)			
Support networks Family Institutions Government Friends		371 (83.2) 50 (11.2) 15 (3.4) 8 (1.8)			

The robust maximum likelihood estimation method was used due to the lack of multivariate normality of the data (Mardia’s coefficient = 802.48). All parameters were significant, with measurement weights greater than .50 (Table 2). The arithmetic mean of the squared measure weights or average variance extracted (AVE) of the strength and self-confidence factor (items 1 to 19) was 47.2%, and its internal consistency was very high (α = .93). The AVE of the social competence factor (items 20 to 27) was 46.8%, and its internal consistency was high (α = .87). The AVE of the family support factor (items 28 to 33) was 55.7% and showed a high internal consistency (α = .89). The AVE of the social support factor (items 34 to 38) was 63.6%, and its internal consistency was high (α = .90). The AVE of the structure factor (items 39 to 43) was 40.5% and had an acceptable internal consistency (α = .76). The overall internal consistency was very high (alpha = .95).

**Table 2:** Descriptive statistics of items and measurement weights of factors for items

Items	Statistics		Measurement weights				
	M	SD	SSC	SC	FS	SS	ST
1. What has happened to me in the past makes me feel confident when facing new challenges.	3.11	0.74	.57				
2. I know where to look for help.	3.05	0.74	.56				
3. I am a strong person.	3.16	0.62	.62				
4. I know very well what I want.	3.14	0.65	.68				
5. I control my life.	2.96	0.69	.59				
6. I like challenges.	2.97	0.73	.60				
7. I work hard to achieve my goals.	3.30	0.56	.73				
8. I am proud of my accomplishments.	3.25	0.62	.72				
9. I know that I have skills.	3.31	0.56	.66				
10. Believing in myself helps me overcome difficult situations.	3.30	0.63	.67				
11. I believe that I am going to succeed.	3.22	0.66	.71				
12. I know how to achieve my goals.	3.08	0.65	.76				
13. No matter what happens, I will always find a solution.	3.26	0.60	.67				
14. My future looks bright.	2.96	0.71	.76				
15. I know that I can solve my personal problems.	3.23	0.57	.75				
16. I am satisfied with myself.	3.13	0.66	.77				
17. I have realistic plans for the future.	3.13	0.66	.70				
18. I trust my decisions.	3.14	0.61	.77				
19. When I am not well, I know that better times will come.	3.28	0.61	.58				
20. I feel comfortable around other people.	2.91	0.69		.66			
21. Making contact with new people is easy for me.	2.86	0.74		.67			
22. It is easy for me to make new friends.	2.83	0.76		.70			
23. Finding a good conversation topic is easy for me.	2.89	0.71		.69			
24. I adapt easily to new situations.	2.89	0.74		.70			
25. Making other people laugh is easy for me.	2.73	0.72		.70			
26. I enjoy being with other people.	2.98	0.64		.69			
27. I know how to start a conversation.	2.90	0.67		.66			
28. I have a good relationship with my family.	3.32	0.69			.82		
29. I enjoy being with my family.	3.47	0.61			.80		
30. In our family, we are loyal to each other.	3.29	0.68			.81		
31. In our family, we enjoy doing activities together.	3.31	0.70			.77		
32. Even in difficult times, our family has an optimistic attitude toward the future.	3.23	0.69			.61		
33. In our family, we agree about what is important in life.	3.25	0.62			.64		
34. I have some friends/relatives who are genuinely concerned about me.	3.26	0.70				.83	
35. I have some friends/relatives who support me.	3.24	0.71				.87	
36. There is always someone who can help me when I need it.	3.26	0.74				.77	
37. I have some friends/relatives who encourage me.	3.25	0.69				.79	
38. I have some friends/relatives who value my skills.	3.16	0.69				.72	
39. Rules and routine make my life easier.	2.81	0.72					.56
40. I maintain my routine even in difficult times.	2.71	0.74					.59
41. I prefer to plan my activities.	2.89	0.68					.65
42. I work better when I have goals.	3.10	0.62					.68
43. I am good at organizing my time.	2.83	0.74					.69

Sample size: N = 446. Method to minimize the discrepancy function: robust maximum likelihood. All parameters are significant at *p* < .001. Descriptive statistics: *M* = Arithmetic mean, and *SD* = Standard deviation. Item response options: 1 = “Strongly disagree”, 2 = “disagree”, 3 = “agree”, and 4 = “strongly agree”. Factors: SSC = Strength and self-confidence, SC = Social competence, FS = Family support, SS = Social support, and ST = Structure

The correlations between the five factors were significant and ranged from moderate (*r* = .41, *p* < .001) to very high (*r* = .74, *p* < .001) (Table 3). The mean shared variance between pairs of factors was 31.5%. Although goodness of fit was rejected by a chi-square test (_S-B_χ^2^_[840, *N* = 446]_ = 1397.91, *p* < .001), the other three indexes showed a good fit to the data: χ^2^/*df* = 1.66, *CFI* = .95, and *.03 RMSEA* = (IC 90%: .02, .04).

**Table 3:** Correlations among factors

Factors	SSC	SC	FS	SS	ST
(SSC) Strength and self-confidence	1				
(SC) Social competence	.74 ***	1			
(FS) Family support	.42 ***	.56 ***	1		
(SS) Social support	.67 ***	.53 ***	.41 ***	1	
(ST) Structure	.63 ***	.47 ***	.45 ***	.63 ***	1

***p < .001 (for a two-tailed Fisher’s test). Sample size: *N* = 446. Method: Robust maximum likelihood estimation

Finally, t-tests did not reveal gender differences in the overall scale score or in the factors (*p* > .05), as shown in Table 4.

**Table 4:** Comparison of means of RESI-M total score and its factors between women and men

RESI-M	Items	Women (*N* = 367)		Men (*N* = 79)		Total sample (*N* = 446)		*t*-test		
		M	SD	M	SD	M	SD	t	df	*p*
Total score	1–43	133.40	17.17	132.99	14.67	133.33	16.74	0.20	444	.842
Strength and self-confidence	1–19	59.92	8.80	60.24	6.85	59.98	8.48	−0.36	139.42	.720
Social competence	20–27	23.06	4.17	22.66	3.98	22.99	4.14	0.78	444	.437
Family support	28–33	19.85	3.33	19.89	2.48	19.86	3.20	−0.11	146.07	.913
Social support	34–38	16.25	2.98	15.84	2.95	16.18	2.97	1.12	444	.263
Structure	39–43	14.33	2.56	14.37	2.28	14.34	2.51	−0.12	444	.904

*N* = Sample size, *M* = Arithmetic mean, *SD* = Standard deviation. Student’s test: t = Value of the test statistic, df = Degrees of freedom (if df = 444, equality of variance is assumed to be tested using Levene’s test, and if df < 444, equality of variance is not assumed, and Welch-Satterthwaite correction is used), and *p* = Probability value for a two-tailed test

## Discussion

The general objective of this study was to validate the RESI-M in a population of family caregivers of children hospitalized with chronic diseases. The initial hypothesis was that the factorial structure in this population would be identical to that detected in the general population in the original validation of that instrument [26]. The data obtained confirmed this hypothesis. The present factor analysis, using the RESI-M, indicates a structure composed of five factors: Strength and Self-Confidence, Social Competence, Family Support, Social Support, and Structure. Overall, the factors showed good internal consistency, with Strength and Self-Confidence being very high; Social Competence, Family Support and Social Support high; and Structure acceptable. These findings are similar to those of Palomar and Gómez [26] and Camacho-Valadez [27] with respect to the dimensions used to assess the construct of resilience in the Mexican general population.

These data indicate that general psychometric properties of the RESI-M found in the general population are also found in a specific subpopulation facing conditions of vulnerability and in which greater resilience must be developed—specifically, a population of caregivers of children suffering from chronic illness. In addition, our study indicates the relevance of using RESI-M over a broader age range (from 18 to 50 years) relative to that employed in the original validation that ranged from 18 to 25 years. Unlike the data obtained by Palomar and Gómez [26], in whose study the women’s social support factor average was higher than the men’s one, our data do not indicate differences between the sexes in the average of any resilience factor. This may be due to the particular conditions to which family caregivers are exposed.

A limitation that warrants attention is the use of non-probability sampling. Thus, the results do not represent estimates of population parameters, an issue that must be addressed in future studies. The sample size was 34 participants below the minimum number of participants, based on the rule of including at least five participants per parameter to be estimated [51]; nevertheless, the number was greater than 400. This number is considered a good sample size when the number of indicators per factor is high (6 or more), measurement weights are high (greater than .50), and few factors exist (five or fewer) [51, 52]. These criteria were satisfied in the case of the five-factor model for the RESI-M tested in the present study. Therefore, the sample size should be considered sufficient for conducting a one-group analysis confirmatory analysis. Among the strengths of this study is the use of robust methods in the confirmatory factorial analysis due to non-compliance with the assumption of multivariate normality.

## Conclusion

This study demonstrated that the model of five correlated factors (strength and self-confidence, social competence, family support, social support, and structure), originally developed for the 43 items constituting the RESI among young adults from the general population, is valid among families of hospitalized children suffering from chronic diseases. In addition, the internal consistency values of the RESI-M and its five factors in this population varied from acceptable to very high and do not require the removal of any item for their improvement. Given that no difference in the averages between women and men was observed, the scale and its factors do not require a different standardization for each sex. Thus, the RESI-M can be considered a valid and reliable instrument for measuring and assessing resilience in family caregivers of children with chronic diseases. These results may contribute to research in the field of family resilience in the context of pediatric disease and to the development and implementation of intervention programs for improving the quality of life and well-being of family caregivers.

## Abbreviations

CFA: Confirmatory Factor Analysis
RESI-M: Measurement Scale of Resilience in Mexicans
